# Paraneoplastic Leukemoid reaction in soft tissue sarcoma: A case report and literature review

**DOI:** 10.1016/j.ijscr.2024.109819

**Published:** 2024-05-31

**Authors:** Garcia-Ortega Dorian Yarih, Hall-Ramírez William Bryan, Ortega Jiménez José Antonio, Melendez-Fernandez Ana Paulina, Caro-Sánchez Claudia, Gabriela Alamilla-García, Luna-Ortiz Kuauhyama

**Affiliations:** a Surgical Oncology, Skin, Soft Tissue & Bone Tumors Department, National Cancer Institute, Mexico City, Mexico; b Surgical Oncology Fellow, Surgical Department, National Cancer Institute, Mexico City, Mexico; c Oncology pathologist National Cancer Institute, Mexico City, Mexico; d Medical Oncology Department, National Cancer Institute, Mexico; e Surgical Oncology Department of Head and Neck Surgery Department, National Cancer Institute, Mexico City, Mexico

**Keywords:** Paraneoplastic leukemoid reactions, Spindle cell sarcoma, Soft tissue sarcoma, Case report

## Abstract

**Introduction:**

Paraneoplastic leukemoid reactions (PLRs) in the context of sarcomas represent a unique clinical entity that poses significant diagnostic challenges and adds valuable insights to the surgical literature. Characterized by an abnormal elevation of white blood cell count, these reactions are often associated with aggressive tumor biology and poor prognosis, emphasizing the need for heightened awareness among clinicians.

**Case presentation:**

A 48-year-old male presented with a rapidly growing, ulcerated tumor on his thigh. Lab tests revealed an extreme leukocytosis with a white blood cell count of 92,860/mm3. Imaging and biopsy confirmed a high-grade spindle cell sarcoma.

**Clinical discussion:**

After excluding other causes of leukocytosis, a PLR secondary to sarcoma was diagnosed. Despite initial antibiotic treatment, leukocytosis persisted, prompting a decision for surgical intervention. The patient underwent successful tumor resection, resulting in a significant decrease in leukocyte count and subsequent stable recovery, supported by adjuvant radiotherapy.

**Conclusion:**

This case underscores the importance of recognizing PLRs in sarcoma patients as they can significantly impact clinical management and prognosis. It highlights the necessity of a multidisciplinary approach for accurate diagnosis and effective treatment. The case contributes to the surgical literature by detailing the diagnostic process and therapeutic interventions in managing such complex presentations, thereby providing key “take-away” lessons on the importance of considering PLRs in the differential diagnosis of leukocytosis in patients with malignancies.

## Highlights

• Highlights the importance of considering PLRs in differential diagnosis for leukocytosis in cancer patients, based on a rare spindle cell sarcoma case.
• Demonstrates the efficacy of a multidisciplinary surgical approach in managing a sarcoma patient with a leukemoid reaction, enhancing outcome prospects.
• Adds crucial knowledge on PLRs in sarcoma, urging further research into their diagnostic and therapeutic implications.

## 1 Introduction

Paraneoplastic leukemoid reactions (PLRs) represent an intriguing and complex facet of the systemic response to malignancies. A leukemoid reaction is a hematological disorder characterized by an exaggerated proliferation of white blood cells, often exceeding 50,000 cells/μL, resembling leukemia but caused by triggers outside the bone marrow, such as infections or drugs, rather than a primary hematological malignancy [1]. When this reaction is related to an underlying neoplasm, it is known as a paraneoplastic leukemoid reaction.

PLRs are rare, with an incidence in solid tumors estimated between 1 % and 10 %. They occur in various malignancies, most notably lung cancer, urothelial carcinomas, pancreatic cancer, skin cancer, and others. Only 10 % of PLR cases are associated with soft tissue sarcomas [2]. Recognizing PLRs is crucial for clinicians to avoid unnecessary diagnostic tests and treatments and to guide the appropriate management of the underlying malignancy. Given the scarcity of information on the clinical features and treatment of PLRs, we present the following case report and literature review.

## 2 Clinical case

We present the case of a 48-year-old male with no known risk factors for soft tissue sarcoma, who presented with a lesion on the posterior aspect of the right thigh, which grew progressively in size. Initial examination revealed a 15 × 15 cm fungating, ulcerated tumor on the posterior side of the right thigh, characterized by abundant necrosis and easy bleeding. A biopsy indicated high-grade spindle cell sarcoma. As part of the diagnostic protocol, the following laboratory results were obtained: Leukocytes 92,860/mm3, Neutrophils 84,800/mm3, Lymphocytes 4600/mm3, Monocytes 3300/mm3, Hemoglobin 7.3 g/dl, Platelets 277,000/mm3, INR 1.15, DHL 285 IU/L. A manual differential count confirmed these leukocyte numbers.

A CT scan of the right thigh revealed a 16 × 16 × 15 cm tumor involving the vastus lateralis and vastus intermedius, with a hyperdense peripheral component and hypodense central areas. Given the tumor's macroscopic characteristics and lab results, antibiotic treatment was initiated. The marked increase in leukocyte count prompted evaluation by the hematology team, who performed peripheral blood smears, noting numerous band neutrophils and signs of pseudo Pelger-Huët anomaly with toxic granulation.

**Fig. 1:**
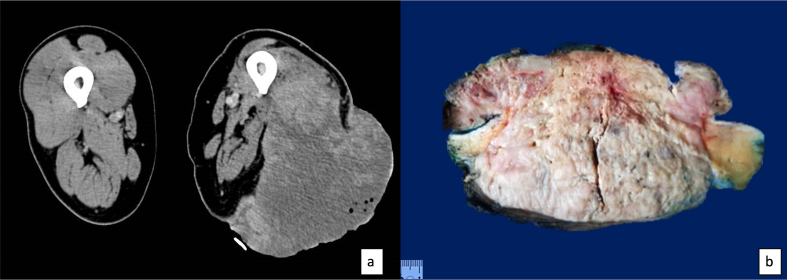
(a) CT of the right thigh, in the axial section, reveals a tumor involving the vastus lateralis and vastus intermedius muscles. The imaging shows a hyperdense peripheral component and hypodense central areas within the tumor. (b) In the cut surface, the neoplasm appears fish-like, white-pink, with hemorrhagic areas.

Despite antibiotic therapy, leukocyte counts remained above 90,000/mm3. Given the high risk of complications and potential intolerance to neoadjuvant treatment due to the leukemoid reaction, surgical intervention was opted for. The patient underwent a wide resection of the lesion without complications (Fig. 1). Postoperatively, leukocyte counts decreased to 26,600/mm3 at 24 h and were followed by further reductions to 7940/mm3 and 5.61/mm3 at 48 h and 7 days, respectively. The patient recovered well and was discharged on the third postoperative day (Table 1).

**Table 1:** White blood cell and neutrophil counts before and after surgery.

Time point	White blood cell count (cells/mm^3^)	Neutrophil count (cells/mm^3^)
Before operation	92,860	84,800
24 hours post-operation	26,600	15,960
48 hours post-operation	7940	4764
7 days post-operation	5610	3366

**Fig. 2:**
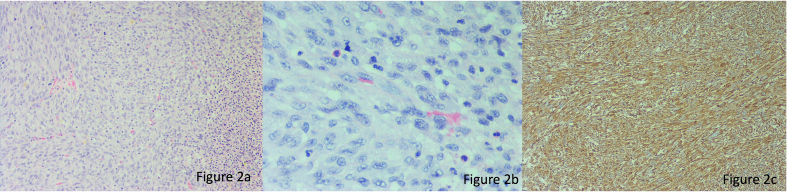
(a, 20x) In the microphotography, we identified a neoplasm of spindle cells, with long bundles, with a solid arrangement. Also, we can identify chronic inflammatory cells (plasma cells, eosinophils, and lymphocytes). (b, 40x) These cells show moderate atypia with vesicular, elongated nuclei, some larger and irregular. (c, 20x) The neoplasm was only diffusely positive for vimentin and CD10.

The surgical specimen was evaluated, revealing a G3 undifferentiated spindle cell sarcoma measuring 17.7x17x10.9 cm, exhibiting 40 % necrosis and no lymph vascular or perineural invasion (Fig. 2). The surgical margins were clear, with distances of 4 cm superiorly, 2 cm inferiorly, 1 cm anteriorly, and 0.5 cm posteriorly, while the surgical bed margin was noted at 0.5 mm. Based on the clinical and pathological findings, the decision was made to proceed with adjuvant chemoradiotherapy. The patient received standard doses of doxorubicin (75 mg/m^2^) and ifosfamide (10 g/m^2^) for soft tissue sarcomas, administered in cycles every three weeks. He tolerated the chemotherapy well, with no reported complications or adverse reactions.

At the 40-month follow-up, the patient is clinically and radiologically free of disease. He is currently under surveillance with an MRI of the limb and chest CT scans, showing no evidence of recurrence or metastasis.

## 3 Discussion

The mechanism underlying PLRs is not entirely understood. Still, tumor cytokine secretion, particularly granulocyte colony-stimulating factor (G-CSF), is believed to play a critical role in inducing this paraneoplastic syndrome. This leukocytosis is usually neutrophilic, but instances of eosinophilic or mixed PLRs have also been reported [3].

Clinically, PLRs represent a significant diagnostic challenge and are made by exclusion of other possible causes of leukocytosis, mainly myeloproliferative neoplasms, but conditions such as infection or iatrogenic (high doses of corticosteroids) leukocytosis must be considered. Recognizing this phenomenon early is paramount to avoid unnecessary diagnostic tests and treatments and to guide the appropriate management of the underlying malignancy [4].

PLRs have been associated with a poor prognosis, usually associated with higher tumor burden and aggressive tumor biology, making identification even more crucial for clinicians [5]. Research into the molecular pathways and therapeutic implications of paraneoplastic leukemoid reactions is ongoing, offering the potential for novel diagnostic and treatment strategies. Being an uncommon entity with scarce information regarding clinical features and treatment we present the following case. Leukemoid reactions, while relatively unusual in the context of sarcomas, are notably significant. This hematological disorder is characterized by a substantial increase in leukocyte count and a pronounced left shift, which occurs without an associated hematologic malignancy [6].

Sarcomas are a diverse group of malignant tumors that arise from connective tissues, including bone, muscle, fat, blood vessels, and other soft tissues. Within these neoplasms, at least 100 histological and molecular subtypes are included, each exhibiting different biological behaviors, and some can trigger leukemoid reactions [7]. Paraneoplastic leukemoid reactions (PLRs) in sarcoma patients represent a diagnostic challenge due to the multitude of secondary causes, such as infections, hemorrhage, hematologic malignancies, use of corticosteroids, and bone necrosis due to metastatic lesions, among others. This makes it a rare phenomenon diagnosed by exclusion [8]. Cut-off limits have been proposed to establish a precise definition, with the most widely accepted being white blood cell (WBC) counts >50,000/μL, excluding secondary etiologies [9].

The correlation between solid tumors like sarcomas and extreme leukocytosis is not well established, partly due to the rarity of the phenomenon. <10 % of sarcoma cases are estimated to present with PLR [8]. PLR in patients with sarcoma could be induced by similar mechanisms to those described in non-hematologic malignancies. This usually happens due to the presence of a non-hematolymphoid cytokine-secreting tumor. The most secreted cytokine is granulocyte colony-stimulating factor (G-CSF); however, other cytokines such as granulocyte monocyte colony-stimulating factor (GM-CSF), interleukin (IL)-1α, IL-1β, IL-3, IL-6, and tumor necrosis factor (TNF)-α may also be secreted, driving extreme neutrophilia [10]. This paracrine effect of cytokines promotes tumor growth and could contribute to the paraneoplastic phenomena observed.

The association between eosinophilia and malignant diseases is well-documented; however, its mechanism and specific relationship with sarcomas are poorly understood [11]. Many hypotheses have been postulated, including the release of protein material from tumor necrosis, the release of chemotactic factors for eosinophils from tumor cells, the seeding of metastatic tumor cells to the bone marrow causing eosinophil production, and the stimulation of bone marrow cells [12]. However, the precise role of eosinophilia as a prognostic factor in sarcoma patients remains uncertain and warrants further research.

In our case, the patient showed a significant reduction in leukocyte and neutrophil levels after the radical resection of the thigh sarcoma, with a decrease of almost 75 % within the first 24 h and normalization by the second postoperative day.

## 4 Conclusion

In conclusion, leukemoid reactions in sarcoma patients represent a diagnostic and therapeutic challenge. A more in-depth understanding of the underlying mechanisms could lead to better disease management and targeted treatment approaches for patients with sarcomas.

## 5 Methods

Work has been reported in line with the SCARE criteria [13].

## Informed consent

Written consent was obtained.

## Ethical approval

This study does not require approval from the ethics committee; written informed consent was obtained from the patient.

## Funding

None.

## Author contribution

G-O DY, HR W, and OJ A: Literature review and manuscript preparation. M-F A and C-S CH: Manuscript review and editing C-S CH, G-O DY and L-O K: Pathology part and diagnostics.

## Guarantor

Dorian Yarih Garcia-Ortega

## Research registration number

NA.

## Conflict of interest statement

None.
